# Evolutionary study of COVID‐19, severe acute respiratory syndrome coronavirus 2 (SARS‐CoV‐2) as an emerging coronavirus: Phylogenetic analysis and literature review

**DOI:** 10.1002/vms3.394

**Published:** 2020-11-18

**Authors:** Alireza Tabibzadeh, Maryam Esghaei, Saber Soltani, Parastoo Yousefi, Mahsa Taherizadeh, Fahimeh Safarnezhad Tameshkel, Mahsa Golahdooz, Mahshid Panahi, Hossein Ajdarkosh, Farhad Zamani, Mohammad Hadi Karbalaie Niya

**Affiliations:** ^1^ Department of Virology Faculty of Medicine Iran University of Medical Sciences Tehran Iran; ^2^ Department of Virology Faculty of Public Health Tehran University of Medical Sciences Tehran Iran; ^3^ Department of Virology Rabies Research Center Pasteur Institute of Iran Tehran Iran; ^4^ Gastrointestinal and Liver Diseases Research Center Iran University of Medical Sciences Tehran Iran

**Keywords:** COVID‐19, evolutionary study, phylogenetic analysis, severe acute respiratory syndrome coronavirus 2 (SARS‐CoV‐2)

## Abstract

Since emerging coronaviruses have always become a human health concern globally especially severe acute respiratory syndrome coronavirus 2 (SARS‐CoV) and Middle East respiratory syndrome coronavirus and a novel coronavirus was introduced in Wuhan, China, in December 2019 (called SARS‐CoV‐2), many researchers focused on its epidemics, virological and clinical features. SARS‐CoV‐2 is classified as *Betacoronaviruses* genus and *Sarbecovirus* subgenus (lineage B). The virus shows a great similarity with SARS‐CoV and bat SARS‐like coronaviruses. In this study, we evaluate SARS‐CoV‐2 virus phylogeny and evolution by using current virus and related sequences.

## 1 INTRODUCTION

Respiratory viral infections are common infections worldwide and have significant associated morbidity, mortality and economic damages (Borchardt & Rolston, 2012). In this regard, common pathogens such as influenza A, B and respiratory syncytial virus, have been joined by emerging viruses such as MERS‐CoV (Middle East respiratory syndrome coronavirus) and the recent Wuhan seafood market pneumonia virus or severe acute respiratory syndrome coronavirus 2 (SARS‐CoV‐2) and have generated notice to the necessity for rapid diagnosis, therapeutic and preventive approaches (Behzadi & Leyva‐Grado, 2019). The coronaviruses have single‐stranded RNA genome which are classified as *Nidovirales* order, *Cornidoviridae* suborder, *Coronaviridae* family, *Orthocoronavirinae* subfamily and are divided into alpha, beta, gamma and delta genera. Based on the 2019 International Committee of Taxonomy of Viruses classification, human associated *Alphacoronaviruses* are included in the *Duvinacovirus* (Human 229E Coronavirus) and *Setracovirus* (NL63 Coronavirus) subgenus. Additionally, *Betacoronaviruses* divide into five different subgenus including *Embecovirus* (also, known as lineage A), *Sarbecovirus* (lineage B), *Hibecovirus*, *Merbecovirus* (lineage C) and *Nobecovirus* (lineage D; https://talk.ictvonline.org/taxonomy/; Luk et al., 2019). Based on this classification, SARS‐CoV and MERS‐CoV are classified in *Sarbecovirus* and *Merbecovirus*, respectively (Luk et al., 2019). Based on phylogenetic study it could also be concluded that the Wuhan Seafood Market Pneumonia Virus, SARS‐CoV‐2 (2019‐Novel Coronavirus) will be classified as *Sarbecovirus* (Chan, Kok, et al., 2020).

**FIGURE 1:**
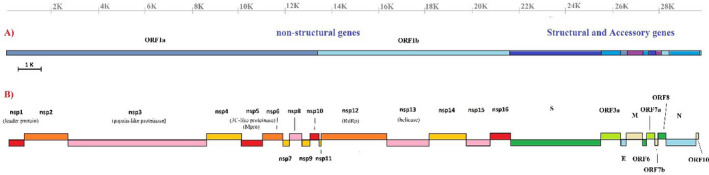
Genome order of SARS‐CoV‐2. (a) Schematic view of the virus genes order; (b) viral encoded proteins and their identified function. SARS‐CoV‐2, severe acute respiratory syndrome coronavirus 2

The coronaviruses encode at least six main ORFs (open reading frames) including *ORF1a/b*, *spike* (*S* protein or surface glycoprotein), *membrane (M)*, *envelope (E)*, *nucleocapsid (N)*, *hemagglutinin (H)* or *hemagglutinin‐esterase (HE)* and also some variable accessory proteins (Figure 1; Chen et al., 2020; Hussain et al., 2005). Conducted studies reveal that SARS‐CoV, MERS‐CoV and SARS‐CoV‐2 contain accessory proteins and ORFs. The accessory ORFs include *3a*, *3b*, *6*, *7a*, *7b*, *8* and *9b* (Luk et al., 2019), while the genome structure in MERS‐CoV contains ORFs *3*, *4a*, *4b*, *5* and *8b* (Boheemen et al., 2012). This genome organization is slightly different in SARS‐CoV‐2 and could be arranged from 5′ to 3′ *ORF 3a*, *6*, *7a*, *7b*, *8* and *10* (Wu et al., 2020).

Epidemics of SARS‐CoV infection as an emerging coronavirus initiated from Guangzhou, China in 2002 by 2003, had spread to 29 countries and lead to 8,089 cases and 9.6% mortality rate. The SARS‐CoV was a recombinant virus from horseshoe bats which was transmitted to the human population through civet cats and was not reported after 2004 (Hui & Zumla, 2019; Hui et al., 2020). The virus phylogeny was comprehensively studied by Luk et al. (2019). Another emerging coronavirus was the MERS‐CoV which was first reported in the Kingdom of Saudi Arabia in 2012 and is still circulating; transmitted to the human population through camels (Azhar et al., 2019). The MERS‐CoV involved 2,465 confirmed cases with an approximate 35% mortality rate (Hui et al., 2020). The recent emerging coronavirus was SARS‐CoV‐2 (2019‐Novel Coronavirus) or Wuhan seafood market pneumonia virus. It was initially reported in some cases presenting unknown pneumonia and associated with the sea food market in Wuhan, China in December 2019 (Bogoch et al., 2020). Based on World Health Organization statistics and situation reports (August 30, 2020), the virus circulated all around the world and led to nearly 25 million confirmed cases and 800,000 deaths worldwide (WHO, 2020). Further investigations established its diagnosis guidelines, virus features, related morbidity and mortality rate (Hui et al., 2020; https://www.who.int/emergencies/diseases/novel‐coronavirus‐2019/technical‐guidance; https://www.who.int/docs/default‐source/coronaviruse/situation‐reports/20200128‐sitrep‐8‐ncov‐cleared.pdf?sfvrsn=8b671ce5_2). In this study, we tried to assess the virus phylogeny and evolution by using current virus related sequences.

## 2 SARS‐COV‐2 CLASSIFICATION AND GENOME ORGANIZATION OF *SARBECOVIRUS*

**FIGURE 2:**
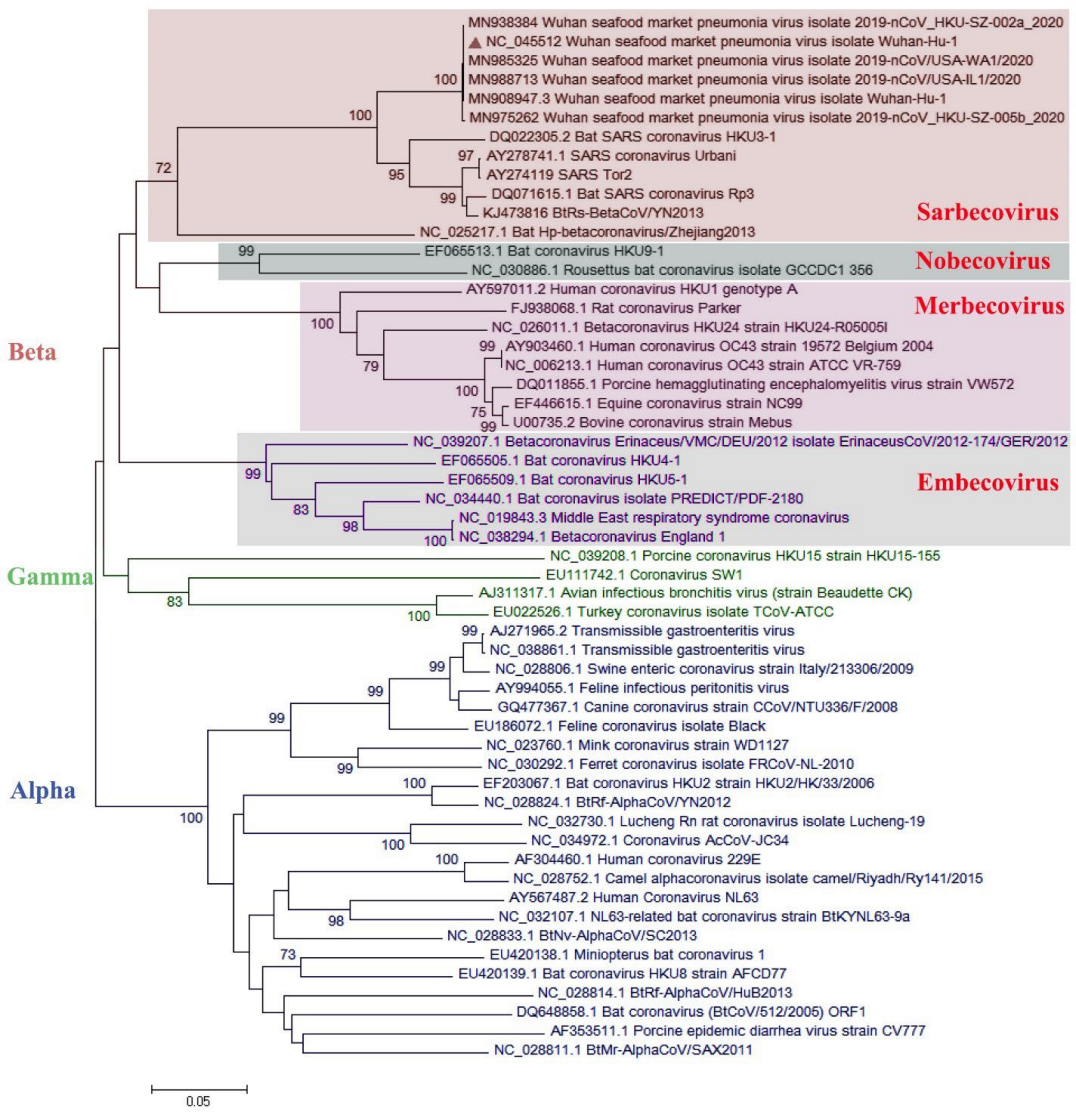
The phylogenetic analysis of 300 bases region of the*RNA‐dependent RNA polymerase*gene of coronaviruses by Neighbour Joining method using 1,000 bootstrap, red triangle showed SARS‐CoV‐2 reference sequence, The scale represents Coronaviruses genus alpha (blue font), gamma (green font) and beta (red font) genus. Beta genus divided into four subgenus (highlighted) included Sarbecovirus, Nobecovirus, Merbecovirus and Embecovirus. SARS‐CoV‐2, severe acute respiratory syndrome coronavirus 2

Most of the virological features and clinical data regarding the SARS‐CoV‐2 have been established by the valuable work of Chan, Yuan, et al. (2020). They conducted a study published in January 2020 in which they reported the SARS‐CoV‐2 classification and genome organization courtesy of Oxford Nanopore Technology sequencing. Their results showed that the virus is related to the bat SARS‐like coronavirus. The virus was also classified as lineage B of *Betacoronavirus*. The genome length is estimated at 29.8 kb having an amount of 38% GC content. The amino acid analysis of SARS‐CoV‐2 showed subunit 1 of the S protein and core receptor binding domain has 66% and 68% similarity with the SARS‐related coronavirus, respectively. The phylogenetic analysis for the 300 base pair region of the virus polymerase gene determined the genera and lineage of this virus (Figure 2). The phylogenic tree showed that the virus located in lineage B, *Betacoronavirus* and is related to the SARS and bat SARS viruses.

The SARS‐CoV‐2 genome structure is mostly similar to the bat‐SARS and other SARS related viruses with slight differences. The genome includes six common ORFs (1a/b, H, S, E, M, and N) and six accessory ORFs (includes 3a, 6, 7a, 7b, 8 and 10; NCBI reference sequence: NC_045512.2). Since the time the complete genome of SARS‐CoV‐2 was published in January 2020 (Chan, Yuan, et al., 2020) many other SARS‐CoV‐2 sequences were reported (MN908947, MN975262, MN988713, MN988669, MN994467, MN994468, MN996527, MN996528, MN996529, MN996530, MN996531, MN997409, MN985325, NC_045512, and etc.). The amino acid sequences alignment for nonstructural proteins of the SARS‐CoV‐2 and bat SARS‐like coronavirus (isolate bat‐SL‐CoVZXC21) showed 88%–99% identity for different proteins, and between SARS‐CoV‐2 and SARS it was 68%–100% (Chan, Kok, et al., 2020). Furthermore, a conducted study by Zhou et al. (2020) indicates that the most similar strain with SARS‐CoV‐2 is bat SARS‐related coronavirus‐RaTG13 (GISAID accession number: EPI_ISL_402131) within 96.2% similarity by the full‐length genome, *RNA‐dependent RNA polymerase (RdRp)* and *spike (S)* genes assessment. The most identical protein between both SARS‐CoV‐2 and SARS‐CoV viruses was helicase (nsp13). SARS‐CoV‐2 protease enzyme also seems to be more similar with the bat SARS‐like coronavirus. The most different amino acid sequence between SARS‐CoV‐2 and SARS‐CoV was ORF 3b. The ORF 3b protein in SARS‐CoV is responsible for the interferon Type I inhibition and induces apoptosis (Liu et al., 2014; Luk et al., 2019). A novel protein in ORF 3b of SARS‐CoV‐2 was also identified which needs further investigation (Chan, Kok, et al., 2020). Recent investigations introduced novel variants of the *RdRp* gene of SARS‐CoV‐2 in patients from Nevada (Hartley et al., 2020). Intrahost virus evolution in cancer patients by Siqueira and colleagues highlights the importance of continued monitoring of the SARS‐CoV‐2 mutations and evolutional patterns (Siqueira et al., 2020). Meanwhile, in a study conducted by Bai et al. (2020) a comprehensive profile from evolution and mutations in SARS‐CoV‐2 was released regarding the assessment of 16,373 genome sequences.

## 3 AN OVERVIEW ON 2019 REPORTED CORONAVIRUS NEW STRAINS

Identification of novel variants and strains in animal coronaviruses, especially bats, is important for understanding the evolution of coronaviruses (Lau et al., 2019). In October 2019 Lau et al. (2019) identified a novel beta coronavirus named *Erinaceus amurensis* Hedgehog Coronavirus HKU31 (Ea‐HedCoV HKU31). The study detected *Erinaceus amurensis* in East China. Lau et al. (2019) also identified a novel *Alphacoronavirus* in horseshoe bats in South China. They named it *Rhinolophus sinicus* bat coronavirus HKU32 (Rs‐BatCoV HKU32) and *Tylonycteris robustula* bat coronavirus HKU33 (Tr‐BatCoV HKU33) which was detected in bats in Guizhou Province, China. In HKU 32 Lau identified a similar protein to SARS ORF 7 and suggested that horseshoe bats were a reservoir for coronavirus epidemics. Additionally, Wang et al. (Lim et al., 2019) discovered a new *Alphacoronavirus* named BtCoV/Rh/YN2012 in the *Rhinolophus* bat with a unique ORF. These studies suggested the important role of bats in the evolution of coronaviruses and the importance of monitoring new strains. The discovery of novel coronavirus strains (for instance, avian infectious bronchitis) also prompted further study (Lim et al., 2019; Xu et al., 2019).

## 4 SARS‐COV‐2 PHYLOGENETIC ANALYSIS AND PROBABLE EVOLUTION

The majority of conducted phylogenetic analysis studied was performed using MEGA X software and the sequences obtained from the NCBI GeneBank (https://www.ncbi.nlm.nih.gov/). Phylogenetic trees were designed by the neighbor‐joining method except for the evolutionary tree which had the maximum likelihood of being the method of choice. The statistical assessment of the trees routinely used the 1,000 replicate bootstrap values (<70 replicates were excluded from the tree branches; Chan, Kok, et al., 2020; Saitou & Nei, 1987).

### 4.1 Envelope

**FIGURE 3:**
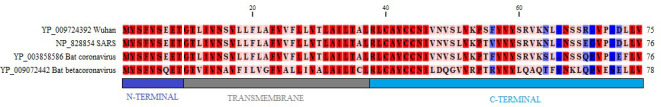
The amino acid sequence of the E protein and three major domains, the amino acid conservation shows by the colours: red colour indicates the most conserve, pink colour indicates less conserve and blue colour indicates gaps or non‐conserve regions

**FIGURE 4:**
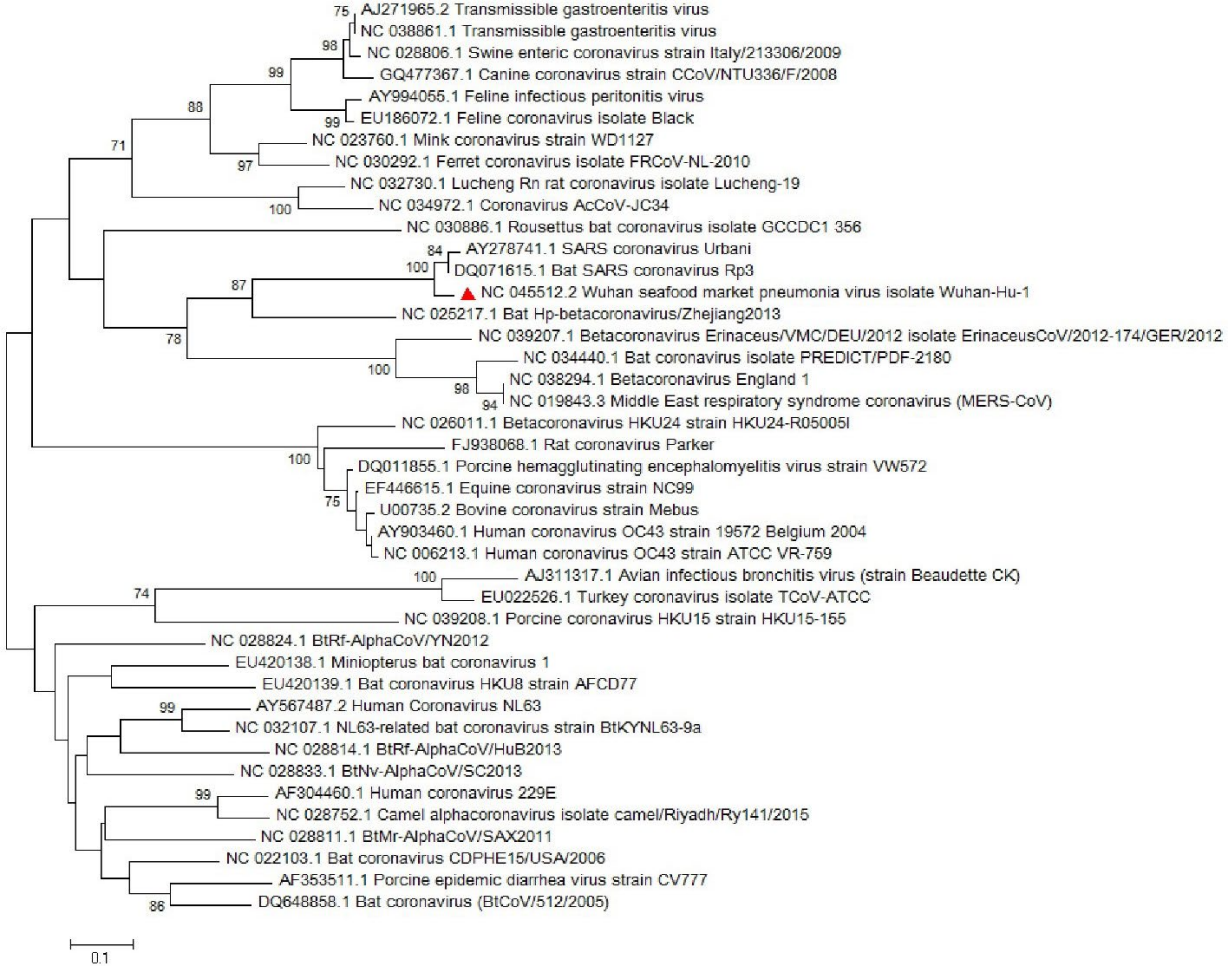
The phylogenetic analysis of 227 bases region of the E gene of coronaviruses by Neighbour Joining method using 1,000 bootstrap. Red triangle showed SARS‐CoV‐2 reference sequence. The scale represents 0.1 substitutions per nucleotide position all of the accession numbers and full name of the strains were listed. SARS‐CoV‐2, severe acute respiratory syndrome coronavirus 2

Conducted researches on the envelope protein of coronaviruses revealed that the protein has short membrane protein and contains three main domains which include N‐terminus, large hydrophobic transmembrane domain (TMD) and C‐terminus (Schoeman & Fielding, 2019). Its domains are illustrated in Figure 3. The E protein is important in virus assembly and is released from the host cell by using the TMD domain and pathogenesis in SARS‐CoV (Hogue & Machamer, 2008; Jimenez‐Guardeno et al., 2014; Schoeman & Fielding, 2019; Ye & Hogue, 2007). Chan, Kok, et al. (2020) showed that the SARS‐CoV‐2 E protein has a 100% similarity to bat‐SL‐CoVZXC21 and 95% similarity to SARS‐CoV. The phylogenetic study for the *E* gene sequence in our study illustrated a great similarity with *sarbecoviruses* which noted the probable importance of the E protein in pathogenesis of SARS‐CoV‐2 (Figure 4).

### 4.2 Spike, Membrane, and Nucleocapsid

**FIGURE 5:**
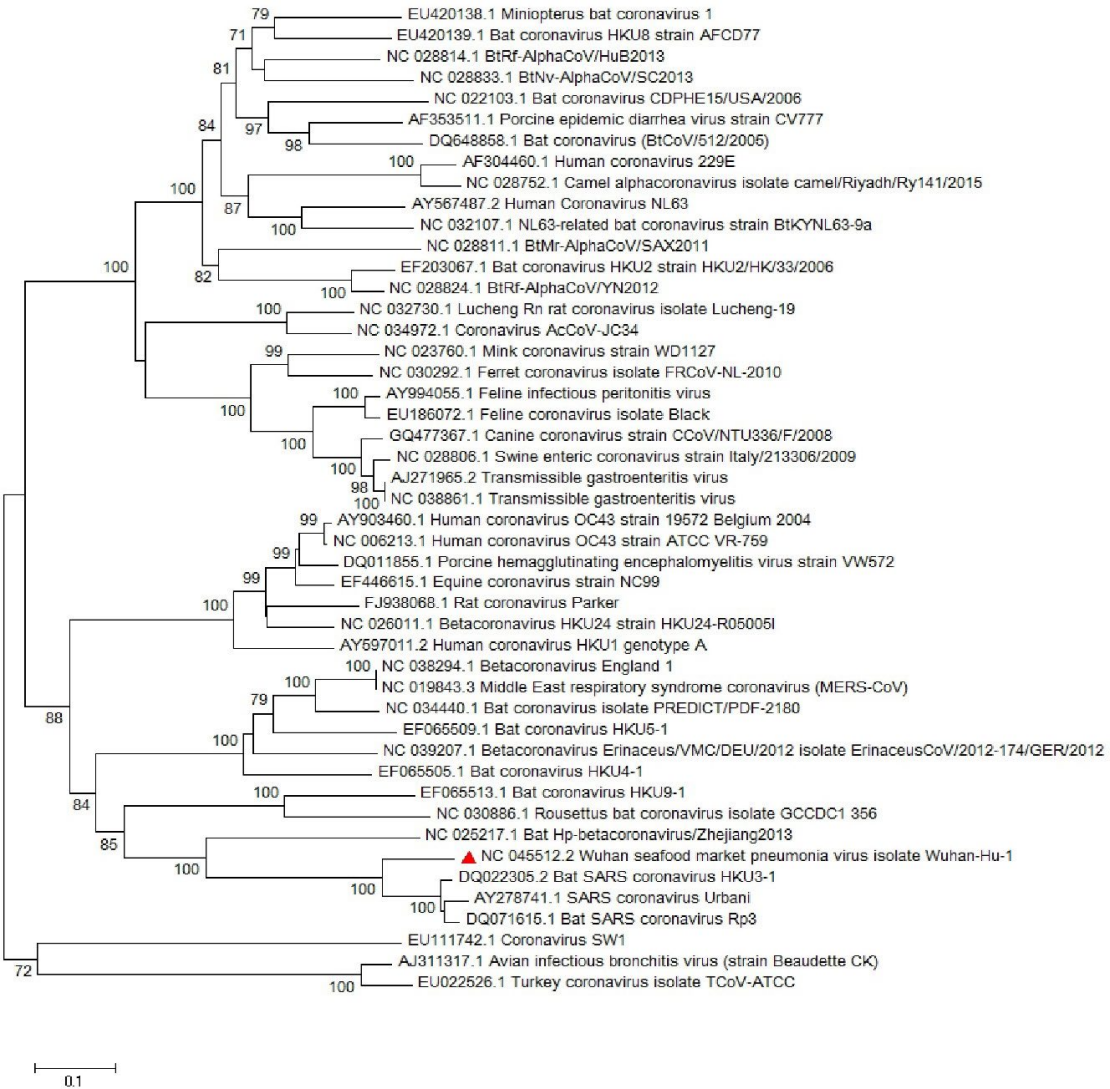
The phylogenetic analysis of 668 bases region of the M gene of coronaviruses by Neighbour Joining method using 1,000 bootstrap, Red triangle showed SARS‐CoV‐2 reference sequence. The scale represents 0.1 substitutions per nucleotide position. All of the accession numbers and full name of the strains were listed. SARS‐CoV‐2, severe acute respiratory syndrome coronavirus 2

**FIGURE 6:**
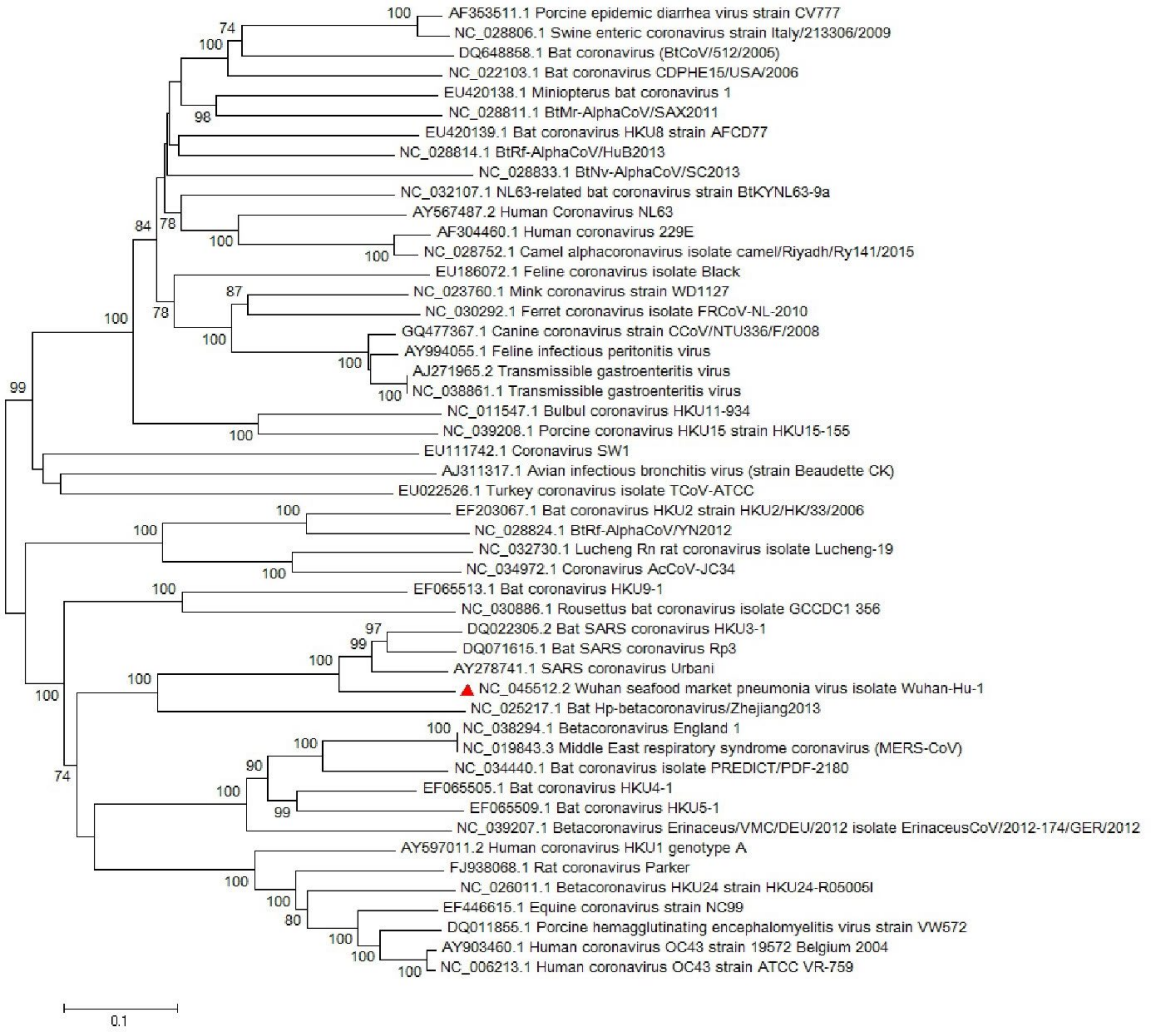
The phylogenetic analysis of 3,821 bases region of the S gene of corona viruses by Neighbour Joining method using 1,000 bootstrap. Red triangle showed SARS‐CoV‐2 reference sequence. The scale represents 0.1 substitutions per nucleotide position. All of the accession numbers and full name of the strains were listed. SARS‐CoV‐2, severe acute respiratory syndrome coronavirus 2

**FIGURE 7:**
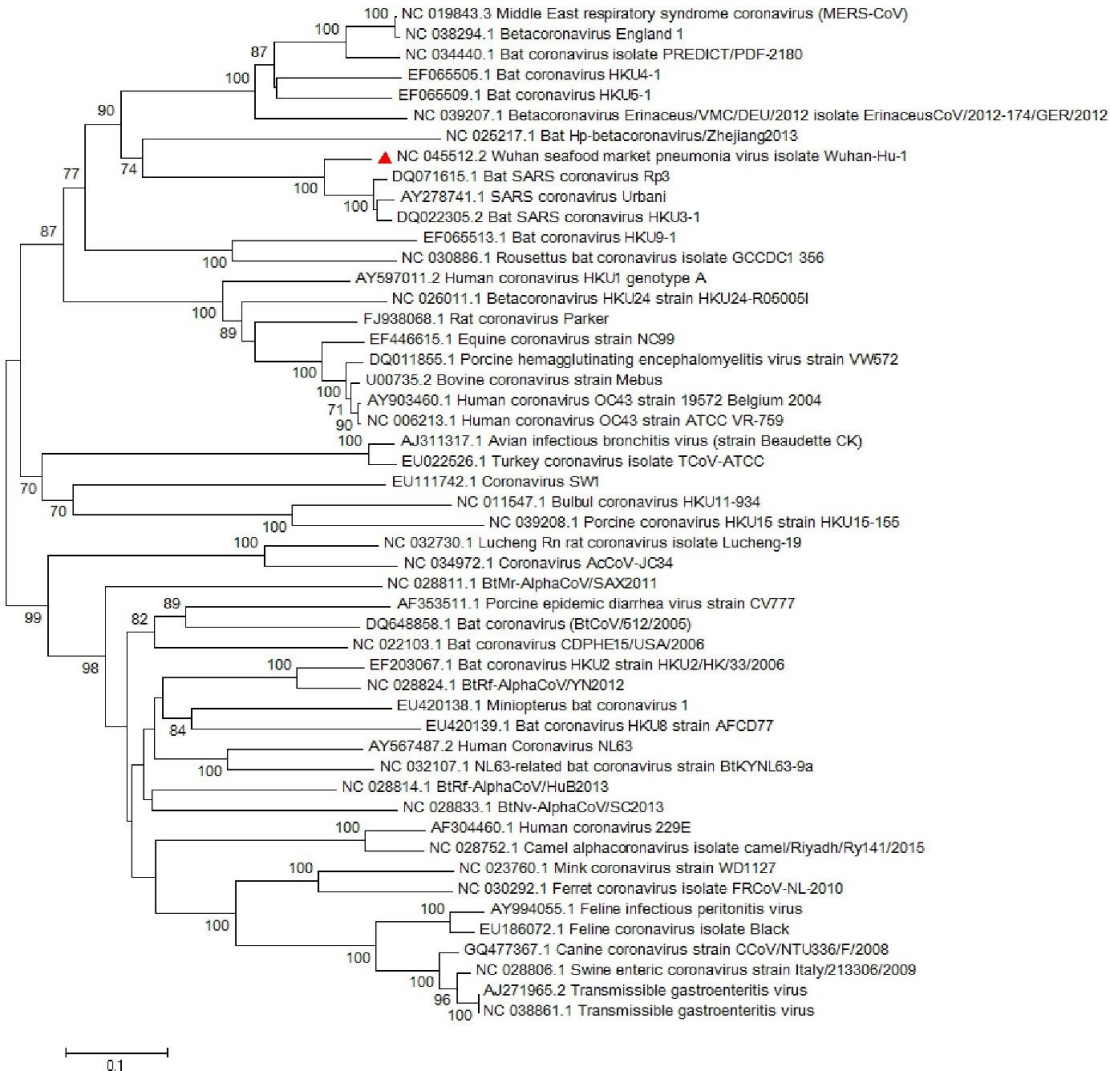
The phylogenetic analysis of 1,259 bases region of the N gene of corona viruses by Neighbor Joining method using 1,000 bootstrap. Red triangle showed SARS‐CoV‐2 reference sequence. The scale represents 0.1 substitutions per nucleotide position. All of the accession numbers and full name of the strains were listed. SARS‐CoV‐2, severe acute respiratory syndrome coronavirus 2

The *M* gene phylogenetic study shows the similarity between the SARS‐CoV‐2 and other *sarbecoviruses* while the main branch shows a similarity with other *betacoronaviruses* such as MERS‐CoV (Figure 5). The *S*, *M* and *N* proteins are responsible for formation of the surface glycoproteins for attachment (Hulswit et al., 2016), inhibition of interferon regulatory protein 3 activation (in SARS‐CoV; Siu et al., 2009) and inhibition of IFN induction in hosts (in SARS‐CoV; Kopecky‐Bromberg et al., 2007), respectively. In this regard, *S*, *M* and *N* genes’ phylogenetic tree was drawn and showed slight differences with other strains (Figures 5, 6, 7).

### 4.3 ORF3

The neighbour‐joining phylogenetic analysis of full length *ORF3* of SARS‐COV‐2 shows the most similarity with bat SARS‐CoV, bat SARS‐CoV HKU‐3 and MERS‐CoV (Figure 10). Conducted study on the MERS‐CoV with deletion on *ORF 3, 4* and *5*, shows that this mutant virus replication and pathogenesis includes anti‐inflammatory responses and are less than the wild‐type virus in human respiratory cells’ culture and animal models (Menachery et al., 2017). *ORF3b* in SARS‐COV‐2 seems to be unique in comparison with SARS‐CoV or SARS‐related‐CoVs. It also revealed that this ORF is important in pathogenesis due to its anti‐IFN activity and despite the differences, suggested that this ORF still could be functional in anti‐IFN activity (Chan et al., 2020).

### 4.4 ORF8

**FIGURE 8:**
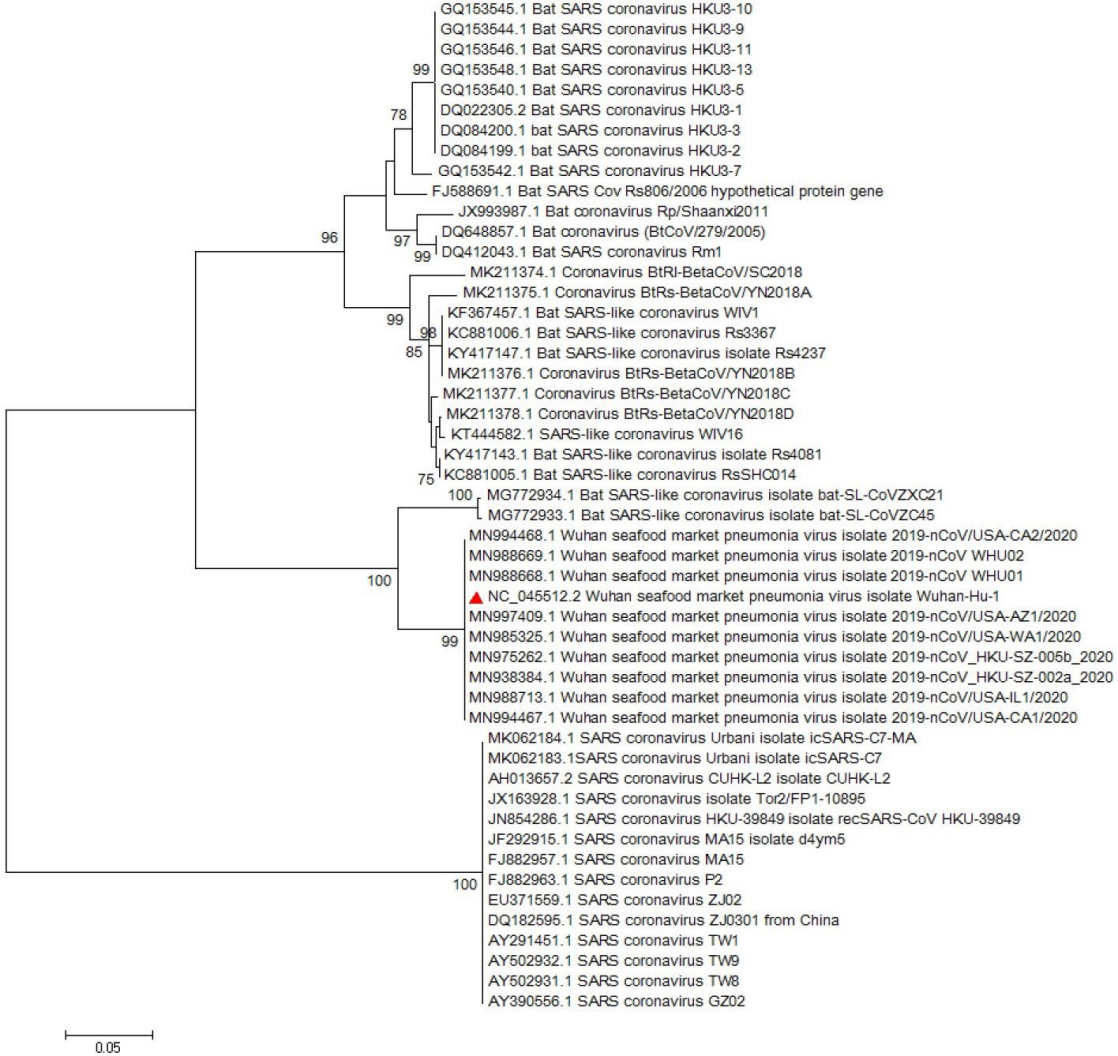
The phylogenetic analysis of 365 bases region of the ORF8 gene of corona viruses by Neighbour Joining method using 1,000 bootstrap. Red triangle showed SARS‐CoV‐2 reference sequence. The scale represents 0.05 substitutions per nucleotide position. All of the accession numbers and full name of the strains were listed. SARS‐CoV‐2, severe acute respiratory syndrome coronavirus 2

**FIGURE 9:**
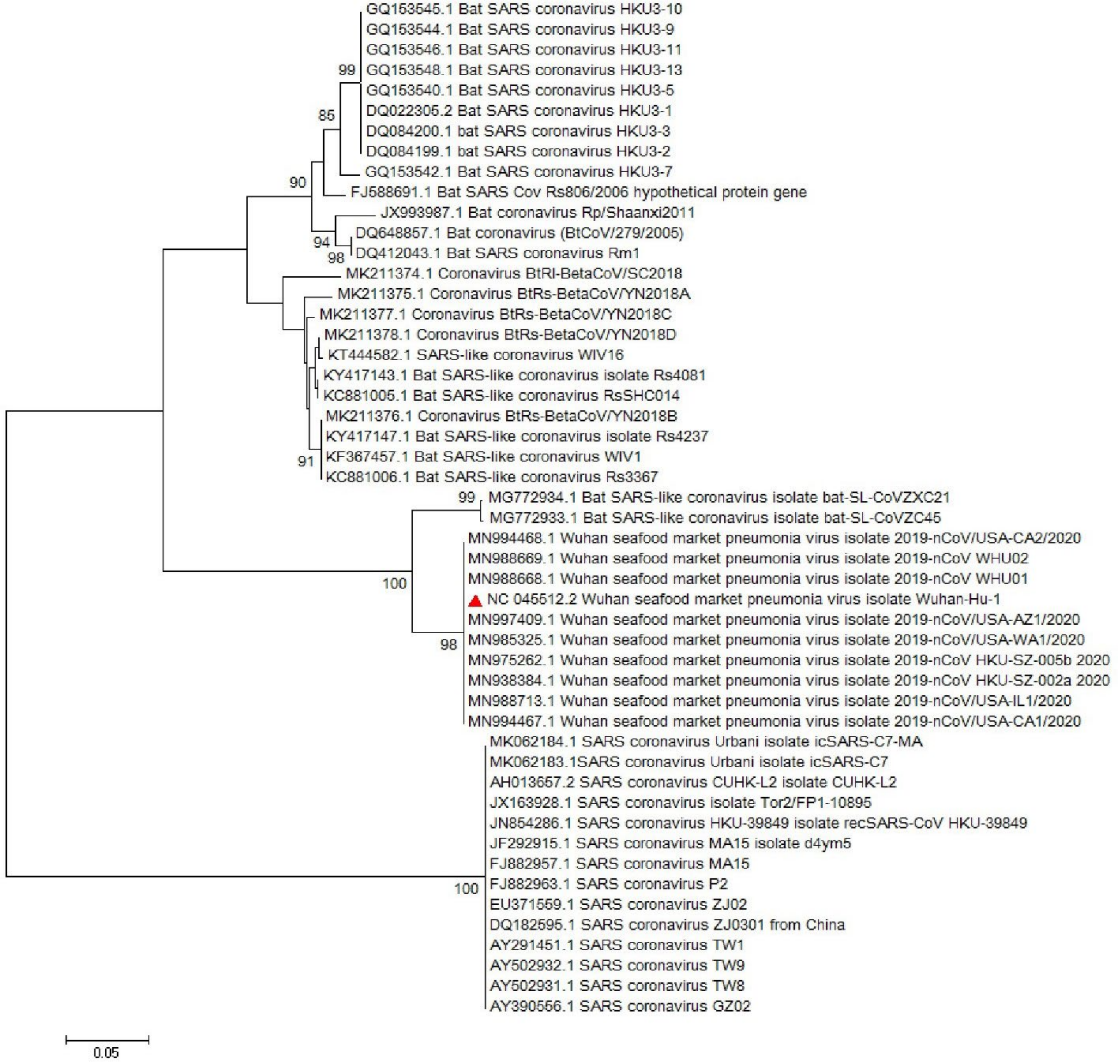
The phylogenetic analysis of 370 bases region of the ORF8 gene of corona viruses by Maximum likelihood method using 1,000 bootstrap. Red triangle showed SARS‐CoV‐2 reference sequence. The scale represents 0.05 substitutions per nucleotide position. All of the accession numbers and full name of the strains were listed. SARS‐CoV‐2, severe acute respiratory syndrome coronavirus 2

Multiple sequence alignment (MSA) of the complete genome from SARS‐CoV‐2 revealed that *ORF8* has unique sequences (Ceraolo & Giorgi, 2020). The phylogenetic study of *ORF8* of the virus shows high similarity with bat SARS‐like coronaviruses (Figures 8 and 9). The importance of *ORF8’s* similarity to SARS‐like coronaviruses and SARS‐CoV is due to the history of the SARS‐CoV epidemic condition. During the start of the SARS‐CoV epidemic, *ORF8* encoded a single protein, while in late periods of the epidemic this ORF encoded two proteins. There is a hypothesis suggested that this change in *ORF8* could be a possible explanation for the SARS‐CoV attenuation and reduction in replication of the virus (Muth et al., 2018; Oostra et al., 2007; Zhang & Liu, 2020).

### 4.5 RdRp (nsp12) and helicase (nsp13)

**FIGURE 10:**
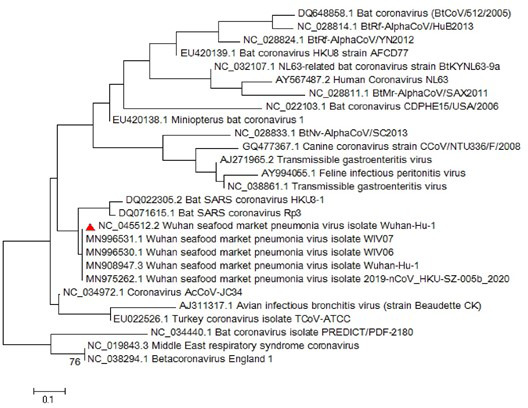
The phylogenetic analysis of 827 bases region of the ORF3 gene of coronaviruses by Neighbour Joining method using 1,000 bootstrap. Red triangle showed SARS‐CoV‐2 reference sequence. The scale represents 0.1 substitutions per nucleotide position. All of the accession numbers and full name of the strains were listed. SARS‐CoV‐2, severe acute respiratory syndrome coronavirus 2

**FIGURE 11:**
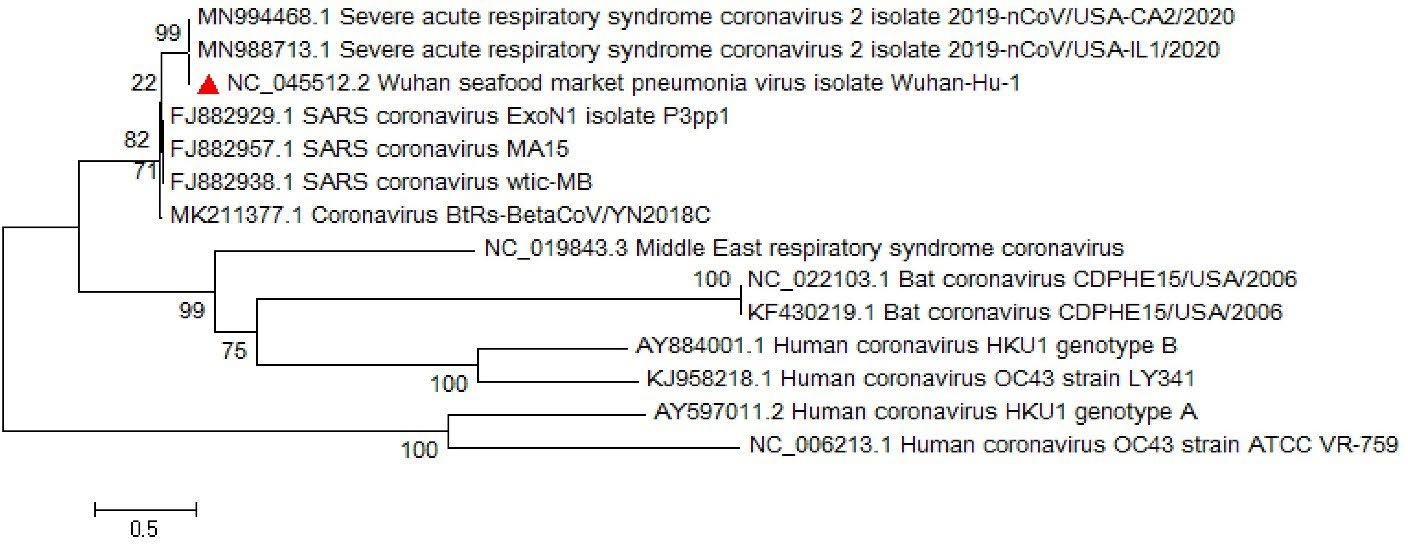
The phylogenetic analysis of 1,802 bases region of the nsp13 (helicase) gene of coronaviruses by Neighbour Joining method using 1,000 bootstrap. Red triangle showed SARS‐CoV‐2 reference sequence. The scale represents 0.5 substitutions per nucleotide position. All of the accession numbers and full name of the strains were listed. SARS‐CoV‐2, severe acute respiratory syndrome coronavirus 2

The *RdRp* sequence of the viruses is used for classification of the viruses into different lineages (Luk et al., 2019) as it discussed in Sections 2 and 5. Furthermore, *nsp13* of the coronaviruses was encoded in the virus helicase (Chen et al., 2020). *Nsp12* or *RdRp* of the virus seems to be important in unwinding the process of the helicase (Jia et al., 2019). Neighbour‐joining analysis shows the helicase of the SARS‐CoV‐2 sequence is similar to the MERS‐CoV, Human Coronavirus OC43, HKU‐1 and some bat coronaviruses as illustrated in Figure 10. The neighbour‐joining of SARS‐CoV‐2 *nsp13* seems to be gene located with sequence heterogeneity between different isolates of SARS‐CoV‐2 (Figure 11), which it highlights the importance of further investigation. The interacting domain of the helicase seems to reside in all coronaviruses and might be a good target for potential therapeutic agents (Jia et al., 2019).

## 5 GEOGRAPHICAL DISTRIBUTION OF SARS‐COV‐2 AND COMPLETE GENOME ALIGNMENT

The first case of SARS‐CoV‐2 in other regions outside China was reported in Thailand in January 2020 (Cheng et al., 2020). In total, 25 million confirmed cases and more than 800,000 deaths worldwide have been recorded due to COVID‐19 as of the 30th of August 2020 (WHO, 2020). The virus evolution studies established by using the 300 base pair reign of *RdRp* clarified the SARS‐CoV and SARS related strains as potent ancestors of the SARS‐CoV‐2. Phylogenetic analysis of *ORF8* gene also proves this context (Figure 9). The SARS‐CoV‐2 strains showed no differences in the time of origin and subsequent evolution based on the phylogenetic tree of *RdRp* gene and *ORF8* by the Maximum Likelihood method (Figure 9).

**FIGURE 12:**
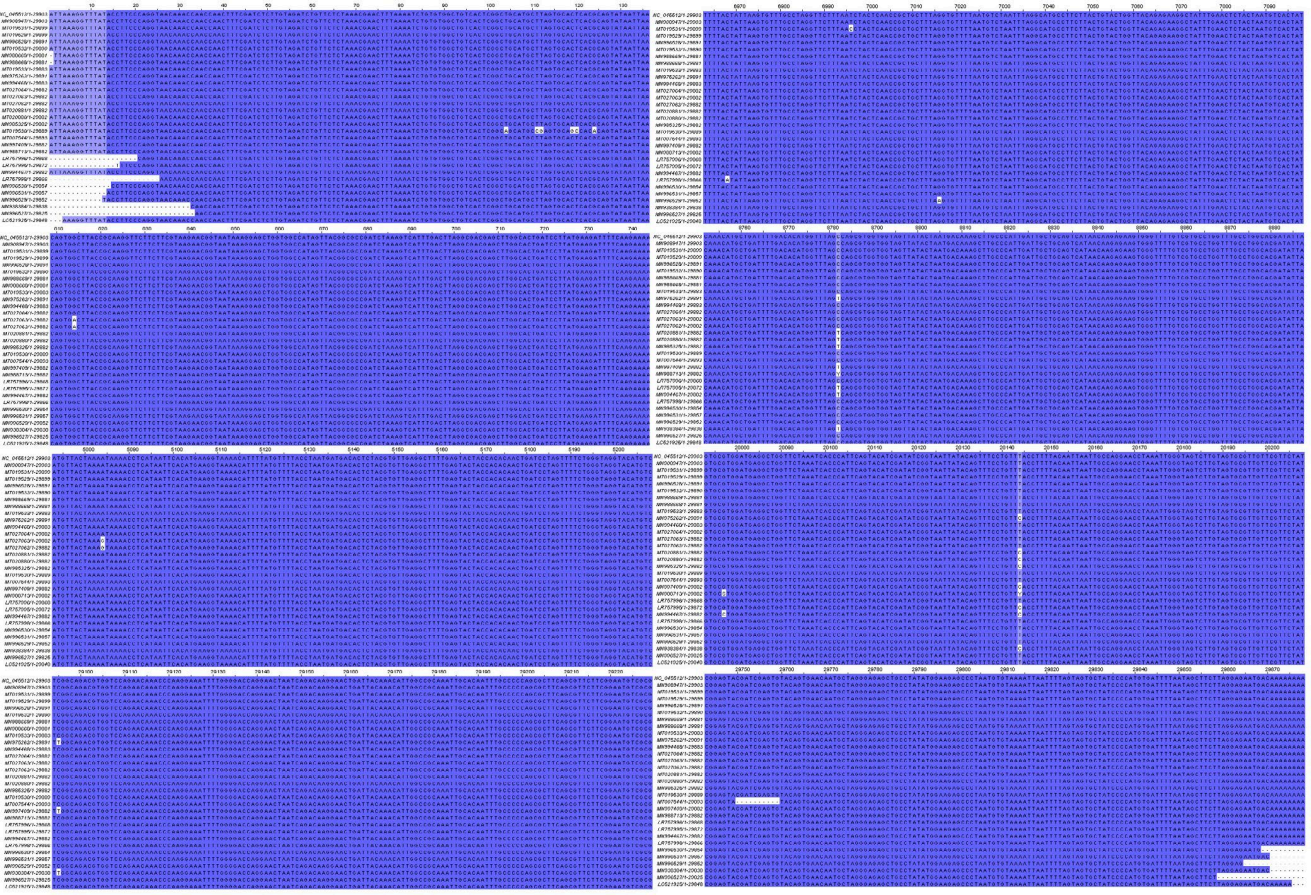
The multiple sequence alignment with Kaligne, the blue sequences shows similarity while the light blue or white sequences shows the differences in full genome alignment of the SARS‐CoV‐2 (screens in figure are truncated and only the highly different positions are illustrated), this sequences mostly obtained from primary full genome sequences in China and USA. SARS‐CoV‐2, severe acute respiratory syndrome coronavirus 2

The MSA for the assessment of the variable nucleotide positions between 31 different complete sequences were performed by the EMBL‐EBI Kaligne online tool and was illustrated in Jalview version 2.10.5 (Figure 12). The MSA shows remarkable differences between different SARS‐CoV‐2 full genome sequences in nucleotide numbers 8,782 and 28,144 which might be located in *ORF1a/b* (nsp‐4) and *ORF8* genes, respectively. Five nucleotide nicks were also seen in 29,750 to 29,760 (*3'UTR*) in one of the Australian isolates (MT007544). The differences in *ORF8* sequence was reported previously which confirms this result (Siu et al., 2009).

## 6 CURRENT THERAPEUTIC OPTIONS FOR SARS‐COV‐2

The history of anti‐protease drugs for the treatment in coronaviruses returns to the SARS‐CoV epidemic (Chu et al., 2004). The use of lopinavir/ritonavir seemed to be potentially beneficial in treating SARS‐CoV patients (Wang et al., 2020). Regardless of being an anti‐HIV drug, Cinanserin (an old 3 chymotrypsin‐like (3C‐like) protease) could be useful in treatment (Zhang et al., 2020). Another drug which also seems to be useful in the treatment of SARS‐CoV‐2 is chloroquine phosphate due to its anti‐inflammatory functions. Furthermore, the chloroquine phosphate function could be effective in increasing endosome pH and preventing virus fusion in the host cell (Gao et al., 2020). In the study conducted by Zhang et al. it was suggested that all of the supportive treatments, coronavirus‐specific treatments and antiviral treatments in infected patients, and using the influenza vaccine in non‐infected people, could be helpful but one of the important matters is the patients’ nutritional status (Zhang et al., 2020). Regarding the nutritional status the role of the Vitamin D still remains controversial (Borst et al., 2011; Zhang et al., 2020). Using the Bacillus Calmette‐Guérin vaccine is recommended (Dara et al., 2020). Recently, the blocking of the virus attachment to ACE‐2 receptors and the employment of Convalescent Plasma Therapy seems to be useful in treatment (Zhang et al., 2020).

## 7 CONCLUSION AND FURTHER PERSPECTIVES

This study reviewed the phylogenetic analysis on the SARS‐CoV‐2 virus, classification and possible ancestors. The current research tried to provide a wide range of the different coronaviruses genus, lineage and strains for a better understanding of the molecular epidemiology of the virus. The SARS‐CoV‐2 is clearly a zoonotic agent and its most possible ancestor is SARS related coronaviruses. The virus classified as *Sarbecovirus* subgenus (or lineage B) of the *betacoronaviruses*. The coronaviruses can induce both upper respiratory tract infections and lower respiratory tract infections in human hosts which clearly shows the importance of these viruses in human diseases as respiratory infection agents (Schoeman & Fielding, 2019). Coronaviruses can be considered zoonotic infectious agents in close human‐animal contact conditions and pass through the species barrier (Schoeman & Fielding, 2019). Further studies for the exact receptor or other virological features of the virus could be suggested. Possible vaccine strategies and effective treatment could be in favour of a better understanding of this virus's possible novel pathogenesis and the virus biology.

## 8 ETHICS

Ethics was approved by Ethical Committee of Iran University of Medical Sciences, Tehran, Iran by the code: IR.IUMS.REC.1398.1327.

## CONFLICT OF INTEREST

None declared.

## AUTHOR CONTRIBUTION

Alireza Tabibzadeh: Data curation; Formal analysis. Maryam Esghaei: Software; Validation. Saber Soltani: Methodology; Visualization. Parastoo Yousefi: Formal analysis; Visualization. Mahsa Taherizadeh: Writing‐original draft; Writing‐review & editing. Fahimeh Safarnezhad Tameshkel: Investigation; Visualization. Mahsa Golahdooz: Investigation; Writing‐review & editing. Mahshid Panahi: Formal analysis; Validation; Writing‐original draft. Hossein Ajdarkosh: Data curation; Supervision; Visualization. Farhad Zamani: Investigation; Resources; Validation. Mohammad Hadi Karbalaie Niya: Conceptualization; Funding acquisition; Project administration; Resources; Writing‐review & editing.

### PEER REVIEW

The peer review history for this article is available at https://publons.com/publon/10.1002/vms3.394.

## References

[vms3394-bib-0001] Azhar, E. I. , Hui, D. S. C. , Memish, Z. A. , Drosten, C. , & Zumla, A. (2019). The middle east respiratory syndrome (MERS). Infectious Disease Clinics of North America, 33(4), 891–905. 31668197 10.1016/j.idc.2019.08.001 PMC7127753

[vms3394-bib-0002] Bai, Y. , Jiang, D. , Lon, J. R. , Chen, X. , Hu, M. , Lin, S. , Chen, Z. , Wang, X. , Meng, Y. , & Dua, H. (2020). Comprehensive evolution and molecular characteristics of a large number of SARS‐CoV‐2 genomes reveal its epidemic trends. International Journal of Infectious Diseases, 100, 164–173. 10.1016/j.ijid.2020.08.066 32866640 PMC7455167

[vms3394-bib-0003] Behzadi, M. A. , & Leyva‐Grado, V. H. (2019). Overview of current therapeutics and novel candidates against influenza, respiratory syncytial virus, and Middle East respiratory syndrome coronavirus infections. Frontiers in Microbiology, 19(10), 1327. 10.3389/fmicb.2019.01327 PMC6594388 31275265

[vms3394-bib-0004] Bogoch, I. I. , Watts, A. , Thomas‐Bachli, A. , Huber, C. , Kraemer, M. U. , & Khan, K. (2020). Pneumonia of unknown aetiology in Wuhan, China: Potential for international spread via commercial air travel. Journal of Travel Medicine, 27(2), taaa008. 31943059 10.1093/jtm/taaa008 PMC7107534

[vms3394-bib-0005] Borchardt, R. A. , & Rolston, K. V. (2012). Respiratory tract infections: Emerging viral pathogens. Journal of the American Academy of Physician Assistants, 25(10), 19–20. 10.1097/01720610-201210000-00005 23115865

[vms3394-bib-0006] Ceraolo, C. , & Giorgi, F. M. (2020). Genomic variance of the 2019‐nCoV coronavirus. Journal of Medical Virology, 92(5), 522–528. 32027036 10.1002/jmv.25700 PMC7166773

[vms3394-bib-0007] Chan, J. F. , Kok, K. H. , Zhu, Z. , Chu, H. , To, K. K. , Yuan, S. , & Yuen, K. Y. (2020). Genomic characterization of the 2019 novel human‐pathogenic coronavirus isolated from a patient with atypical pneumonia after visiting Wuhan. Emerging Microbes & Infections, 9(1), 221–236. 31987001 10.1080/22221751.2020.1719902 PMC7067204

[vms3394-bib-0008] Chan, J. F. , Yuan, S. , Kok, K. H. , To, K. K. , Chu, H. , Yang, J. , Xing, F. , Liu, J. , Yip, C. C. , Poon, R. W. , & Tsoi, H. W. (2020). A familial cluster of pneumonia associated with the 2019 novel coronavirus indicating person‐to‐person transmission: A study of a family cluster. The Lancet, 395(10223), 514–523. 10.1016/S0140-6736(20)30154-9 PMC7159286 31986261

[vms3394-bib-0009] Chen, Y. , Liu, Q. , & Guo, D. (2020). Emerging coronaviruses: Genome structure, replication, and pathogenesis. Journal of Medical Virology, 92(4), 418–423. 31967327 10.1002/jmv.25681 PMC7167049

[vms3394-bib-0010] Cheng, V. C. , Wong, S. C. , To, K. K. , Ho, P. L. , & Yuen, K. Y. (2020). Preparedness and proactive infection control measures against the emerging novel coronavirus in China. Journal of Hospital Infection, 104(3), 254–255. 10.1016/j.jhin.2020.01.010 PMC7134450 31962139

[vms3394-bib-0011] Chu, C. M. , Cheng, V. C. , Hung, I. F. , Wong, M. M. , Chan, K. H. , Chan, K. S. , Kao, R. Y. , Poon, L. L. , Wong, C. L. , Guan, Y. , & Peiris, J. S. (2004). Role of lopinavir/ritonavir in the treatment of SARS: Initial virological and clinical findings. Thorax, 59(3), 252–256. 14985565 10.1136/thorax.2003.012658 PMC1746980

[vms3394-bib-0012] Dara, M. , Sotgiu, G. , Reichler, M. , Chiang, C. , Chee, C. , & Migliori, G. (2020). New diseases and old threats: Lessons from tuberculosis for the COVID‐19 response. International Journal of Tuberculosis and Lung Disease, 24, 544–545. 10.5588/ijtld.20.0151 32398212

[vms3394-bib-0013] de Borst, M. H. , Vervloet, M. G. , ter Wee, P. M. , & Navis, G. (2011). Cross talk between the renin‐angiotensin‐aldosterone system and vitamin D‐FGF‐23‐klotho in chronic kidney disease. Journal of the American Society of Nephrology, 22(9), 1603–1609. 21852584 10.1681/ASN.2010121251 PMC3171931

[vms3394-bib-0014] Gao, J. , Tian, Z. , & Breakthrough, Y. X. (2020). Chloroquine phosphate has shown apparent efficacy in treatment of COVID‐19 associated pneumonia in clinical studies. Bioscience Trends. 10.5582/bst.2020.01047 32074550

[vms3394-bib-0015] Hartley, P. , Tillett, R. L. , Xu, Y. , AuCoin, D. P. , Sevinsky, J. R. , Gorzalski, A. et al (2020). Genomic surveillance revealed prevalence of unique SARS‐CoV‐2 variants bearing mutation in the RdRp gene among Nevada patients. medRxiv. 10.1101/2020.08.21.20178863 PMC7891100 33820739

[vms3394-bib-0016] Hogue, B. G. , & Machamer, C. E. (2008). Coronavirus structural proteins and virus assembly. Nidoviruses, 179–200. 10.1128/9781555815790.ch12

[vms3394-bib-0017] Hui, D. , Madani, T. , Ntoumi, F. , Kock, R. , Dar, O. , Ippolito, G. et al (2020). The continuing 2019‐nCoV epidemic threat of novel coronaviruses to global health—The latest 2019 novel coronavirus outbreak in Wuhan, China. International Journal of Infectious Diseases, 91, 264. 31953166 10.1016/j.ijid.2020.01.009 PMC7128332

[vms3394-bib-0018] Hui, D. S. C. , & Zumla, A. (2019). Severe acute respiratory syndrome: Historical, epidemiologic, and clinical features. Infectious Disease Clinics of North America, 33(4), 869–889. 31668196 10.1016/j.idc.2019.07.001 PMC7127569

[vms3394-bib-0019] Hulswit, R. J. , de Haan, C. A. , & Bosch, B. J. (2016). Coronavirus spike protein and tropism changes. Advances in Virus Research, 96, 29–57. 27712627 10.1016/bs.aivir.2016.08.004 PMC7112277

[vms3394-bib-0020] Hussain, S. , Chen, Y. , Yang, Y. , Xu, J. , Peng, Y. , Wu, Y. et al (2005). Identification of novel subgenomic RNAs and noncanonical transcription initiation signals of severe acute respiratory syndrome coronavirus. Journal of Virology, 79(9), 5288–5295. 15827143 10.1128/JVI.79.9.5288-5295.2005 PMC1082772

[vms3394-bib-0021] Jia, Z. , Yan, L. , Ren, Z. , Wu, L. , Wang, J. , Guo, J. et al (2019). Delicate structural coordination of the severe acute respiratory syndrome coronavirus Nsp13 upon ATP hydrolysis. Nucleic Acids Research, 47(12), 6538–6550. 31131400 10.1093/nar/gkz409 PMC6614802

[vms3394-bib-0022] Jimenez‐Guardeno, J. M. , Nieto‐Torres, J. L. , DeDiego, M. L. , Regla‐Nava, J. A. , Fernandez‐Delgado, R. , Castaño‐Rodriguez, C. et al (2014). The PDZ‐binding motif of severe acute respiratory syndrome coronavirus envelope protein is a determinant of viral pathogenesis. PLoS Path, 10(8). 10.1371/journal.ppat.1004320 PMC4133396 25122212

[vms3394-bib-0023] Kopecky‐Bromberg, S. A. , Martínez‐Sobrido, L. , Frieman, M. , Baric, R. A. , & Palese, P. (2007). Severe acute respiratory syndrome coronavirus open reading frame (ORF) 3b, ORF 6, and nucleocapsid proteins function as interferon antagonists. Journal of Virology, 81(2), 548–557. 17108024 10.1128/JVI.01782-06 PMC1797484

[vms3394-bib-0024] Lau, S. K. P. , Wong, A. C. P. , Zhang, L. , Luk, H. K. H. , Kwok, J. S. L. , Ahmed, S. S. et al (2019). Novel bat alphacoronaviruses in Southern China support Chinese horseshoe bats as an important reservoir for potential novel coronaviruses. Viruses, 11(5). 10.3390/v11050423 PMC6563315 31067830

[vms3394-bib-0025] Lim, X. F. , Lee, C. B. , Pascoe, S. M. , How, C. B. , Chan, S. , Tan, J. H. et al (2019). Detection and characterization of a novel bat‐borne coronavirus in Singapore using multiple molecular approaches. The Journal of General Virology, 100(10), 1363–1374. 31418677 10.1099/jgv.0.001307 PMC7079695

[vms3394-bib-0026] Liu, D. X. , Fung, T. S. , Chong, K.‐K.‐L. , Shukla, A. , & Hilgenfeld, R. (2014). Accessory proteins of SARS‐CoV and other coronaviruses. Antiviral Research, 109, 97–109. 24995382 10.1016/j.antiviral.2014.06.013 PMC7113789

[vms3394-bib-0027] Luk, H. K. , Li, X. , Fung, J. , Lau, S. K. , & Woo, P. C. (2019). Molecular epidemiology, evolution and phylogeny of SARS coronavirus. Infection, Genetics and Evolution, 1(71), 21–30. 10.1016/j.meegid.2019.03.001 PMC7106202 30844511

[vms3394-bib-0028] Menachery, V. D. , Mitchell, H. D. , Cockrell, A. S. , Gralinski, L. E. , Yount, B. L. , Graham, R. L. et al (2017). MERS‐CoV accessory ORFs play key role for infection and pathogenesis. MBio, 8(4), e00665‐17. 28830941 10.1128/mBio.00665-17 PMC5565963

[vms3394-bib-0029] Muth, D. , Corman, V. M. , Roth, H. , Binger, T. , Dijkman, R. , Gottula, L. T. et al (2018). Attenuation of replication by a 29 nucleotide deletion in SARS‐coronavirus acquired during the early stages of human‐to‐human transmission. Scientific Reports, 8(1), 1–11. 30310104 10.1038/s41598-018-33487-8 PMC6181990

[vms3394-bib-0030] Oostra, M. , De Haan, C. A. , & Rottier, P. J. (2007). The 29‐nucleotide deletion present in human but not in animal severe acute respiratory syndrome coronaviruses disrupts the functional expression of open reading frame 8. Journal of Virology, 81(24), 13876–13888. 17928347 10.1128/JVI.01631-07 PMC2168875

[vms3394-bib-0031] Saitou, N. , & Nei, M. (1987). The neighbor‐joining method: A new method for reconstructing phylogenetic trees. Molecular Biology and Evolution, 4(4), 406–425. 3447015 10.1093/oxfordjournals.molbev.a040454

[vms3394-bib-0032] Schoeman, D. , & Fielding, B. C. (2019). Coronavirus envelope protein: Current knowledge. Virology Journal, 16(1), 69. 31133031 10.1186/s12985-019-1182-0 PMC6537279

[vms3394-bib-0033] Siqueira, J. D. , Goes, L. R. , Alves, B. M. , de Carvalho, P. S. , Cicala, C. , Arthos, J. et al (2020). SARS‐CoV‐2 genomic and quasispecies analyses in cancer patients reveal relaxed intrahost virus evolution. bioRxiv. 10.1101/2020.08.26.267831 PMC7928633 33738124

[vms3394-bib-0034] Siu, K. L. , Kok, K. H. , Ng, M. H. , Poon, V. K. , Yuen, K. Y. , Zheng, B. J. et al (2009). Severe acute respiratory syndrome coronavirus M protein inhibits type I interferon production by impeding the formation of TRAF3.TANK.TBK1/IKKepsilon complex. The Journal of Biological Chemistry, 284(24), 16202–16209. 19380580 10.1074/jbc.M109.008227 PMC2713514

[vms3394-bib-0036] van Boheemen, S. , de Graaf, M. , Lauber, C. , Bestebroer, T. M. , Raj, V. S. , Zaki, A. M. et al (2012). Genomic characterization of a newly discovered coronavirus associated with acute respiratory distress syndrome in humans. MBio, 3(6), e00473‐12. 23170002 10.1128/mBio.00473-12 PMC3509437

[vms3394-bib-0037] Wang, Z. , Chen, X. , Lu, Y. , Chen, F. , & Zhang, W. (2020). Clinical characteristics and therapeutic procedure for four cases with 2019 novel coronavirus pneumonia receiving combined Chinese and Western medicine treatment. Bioscience Trends. 10.5582/bst.2020.01030 32037389

[vms3394-bib-0038] WHO . (2020). Situation reports. Retrieved from https://www.who.int/emergencies/diseases/novel‐coronavirus‐2019/situation‐reports

[vms3394-bib-0039] Wu, A. , Peng, Y. , Huang, B. , Ding, X. , Wang, X. , Niu, P. , Meng, J. , Zhu, Z. , Zhang, Z. , Wang, J. , & Sheng, J. (2020). Genome composition and divergence of the novel coronavirus (2019‐nCoV) originating in China. Cell Host & Microbe. 10.1016/j.chom.2020.02.001 PMC7154514 32035028

[vms3394-bib-0040] Xu, L. , Ren, M. , Sheng, J. , Ma, T. , Han, Z. , Zhao, Y. et al (2019). Genetic and biological characteristics of four novel recombinant avian infectious bronchitis viruses isolated in China. Virus Research, 263, 87–97. 30641197 10.1016/j.virusres.2019.01.007 PMC7185608

[vms3394-bib-0041] Ye, Y. , & Hogue, B. G. (2007). Role of the coronavirus E viroporin protein transmembrane domain in virus assembly. Journal of Virology, 81(7), 3597–3607. 17229680 10.1128/JVI.01472-06 PMC1866030

[vms3394-bib-0042] Zhang, H. , Penninger, J. M. , Li, Y. , Zhong, N. , & Slutsky, A. S. (2020). Angiotensin‐converting enzyme 2 (ACE2) as a SARS‐CoV‐2 receptor: Molecular mechanisms and potential therapeutic target. Intensive Care Medicine 46(4), 586–590. 32125455 10.1007/s00134-020-05985-9 PMC7079879

[vms3394-bib-0043] Zhang, L. , & Liu, Y. (2020). Potential interventions for novel coronavirus in China: A Systematic Review. Journal of Medical Virology. 10.1002/jmv.25707 PMC7166986 32052466

[vms3394-bib-0044] Zhou, P. , Yang, X.‐L. , Wang, X.‐G. , Hu, B. , Zhang, L. , Zhang, W. et al (2020). A pneumonia outbreak associated with a new coronavirus of probable bat origin. Nature, 579(7798), 270–273. 32015507 10.1038/s41586-020-2012-7 PMC7095418

